# Supplementary material for: Skeletal Torsion Tunneling and Methyl Internal Rotation: The Coupled Large Amplitude Motions in Phenyl Acetate
Source: Molecules. 2022 Apr 23;27(9):2730. doi: 10.3390/molecules27092730 (PMC9105391; doi:10.3390/molecules27092730)
Supplement: Supplementary file 1 [file molecules-27-02730-s001.zip › molecules-1677120-supplementary.pdf]

Supporting Information

Skeletal torsion tunneling and methyl internal rotation: The coupled large amplitude motions in phenyl acetate

Lynn Ferres [1], Luca Evangelisti [2], Assimo Maris [2], Sonia Melandri [2], Walther Caminati [2,*], Wolfgang Stahl [1,**] and Ha Vinh Lam Nguyen [3,4,*]

[1] *RWTH Aachen University, D-52074 Aachen, Germany*

[2] *Dipartimento di Chimica "G. Ciamician", Università di Bologna, Italy*

[3] *Univ Paris Est Creteil and Université Paris Cité, CNRS, LISA, F-94010 Créteil, France*

[4] *Institut Universitaire de France (IUF), F-75231 Paris, France*

* *Correspondence*: *lam.nguyen@lisa.ipsl.fr, walther.caminati@unibo.it*

** *Deceased*

Table S1. Fourier expansion of the potential energy curve of phenyl acetate calculated at the MP2/6-311++G(d,p) level of theory given in Figure 3. The data were obtained by rotating phenyl ring about the O_{12}–C_{13} bond by varying the dihedral angle α = ∠(C_{13}-O_{12}-C_3-C_2) in a grid of 10°, while all other molecular parameters were optimized. The potential is expanded as:

$$V(\beta) = b_0 + \sum_{n=1}^{7} b\ \cos(n\beta);.$$

Fourier expansion of the potential energy curves given in Figure 4 calculated at the MP2/6-311++G(d,p) level of theory, obtained by rotating the methyl group of phenyl acetate about the C_{13}–C_{14} bond (variation of γ = ∠(O_{15}-C_{13}-C_{14}-H_{16})). The potential is expanded as

$$V(\alpha) = a_0 + \sum_{n=1}^{3} a_n \cos(n\alpha).$$

	Hartree	cm^{-1}
b_0	–448.9579885	
b_2	0.0017257	378.75
b_4	0.0006207	136.23
b_6	0.0000659	14.46
b_8	0.0000508	11.15
b_{10}	0.0000167	3.67
a_0	–448.9579885	
a_3	–0.0002567	−56.34
a_6	0.0000301	6.61
a_9	−0.0000011	−0.24

Table S2. Nuclear coordinates in the principal inertial axes of conformer I_b of phenyl acetate calculated at the MP2/6-311++G(d,p) level of theory. The atoms are numbered according to Figure 2.

	I_b		
	a /Å	*b* /Å	*c* /Å
C_1	2.129450	−1.241971	−0.259741
C_2	0.771955	−1.042572	−0.530908
C_3	0.224578	0.225051	−0.333328
C_4	0.999498	1.295734	0.109160
C_5	2.357977	1.086871	0.373650
C_6	2.924949	−0.179501	0.187634
H_7	2.566113	−2.225941	−0.406045
H_8	0.140800	−1.855184	−0.877385
H_9	0.536384	2.268731	0.243770
H_{10}	2.971502	1.914671	0.718058
H_{11}	3.979207	−0.339289	0.394552
O_{12}	−1.114437	0.469936	−0.653461
C_{13}	−2.048428	−0.090306	0.185998
C_{14}	−3.430315	0.246504	−0.304588
O_{15}	−1.765125	−0.742914	1.159689
H_{16}	−3.549137	1.331532	−0.349219
H_{17}	−3.565594	−0.146130	−1.315149
H_{18}	−4.168358	−0.185816	0.369378

Table S3. The Fourier coefficients of the two-dimensional potential energy surface calculated at the MP2/6-311++G(d,p) level of theory by varying α and γ in a grid of 10° ,while all other parameters were optimized (Figure 5).

N°	Fourier term	Coefficient / Hartree	Coefficient / cm^{-1}
1	1	–458.9564929	/
2	cos(2α)	0.0017806	390.80
3	cos(4α)	0.0006556	143.89
4	cos(6α)	0.0000692	15.19
5	cos(3γ)	–0.0002707	–59.41
6	cos(6γ)	0.0000358	7.86
7	cos(2α)· cos(3γ)	–0.0000171	–3.75
8	cos(2α)· cos(6γ)	0.0000098	2.15
9	sin(2α)· sin(3γ)	–0.0000606	–13.30
10	cos(4α)· cos(3γ)	–0.0000125	–2.74
11	sin(4α)· sin(3γ)	–0.0000554	–12.16
12	sin(6α)· sin(3γ)	–0.0000249	–5.46

Table S4: Fitted molecular parameters of the A0 and A1 state lines of phenyl acetate using the program *spfit*. The experimental accuracy of the microwave (MW) lines is 2 kHz; that of the millimeterwave (mm) lines is 50 kHz; Observed frequencies ($\nu_{obs.}$, in MHz) of 80 rotational transitions of the A0 species (Fit A0 in Table S-4a), those of 20 A1 species transitions (Fit A0/A1 MW in Table S-4a), as well as those measured in the millimeterwave range (Fit A0/A1 mmw in Table S-4a) of phenyl acetate. $\nu_{Obs.} - \nu_{Calc.}$ values (in MHz) are obtained after a fit with the program *spfit*.

**Par.**[a]	**Unit**	**Fit A0**	**Fit A0/A1 MW**		**Fit A0/A1 MW+mmw**	
A	MHz	3636.711(15)	3637.77426(26)	3640.91673(57)	3637.78574(22)	3640.89497(27)
B	MHz	802.815(15)	803.889579(74)	803.38861(17)	803.889288(63)	803.39054(16)
C	MHz	749.905(15)	750.979441(79)	749.94790(38)	750.978551(62)	749.94787(31)
D_J	kHz	0.1279(56)	0.2379(32)	−0.0696(48)	−0.2265(12)	0.0480(24)
D_{JK}	kHz	---	---	6.245(45)	−1.445(13)	−3.829(16)
D_K	kHz	2.64(27)	---	0.01132(52)	−0.924 (15)	1.350(22)
d_1	kHz		---	−0.0550(19)	---	0.0394(12)
d_2	kHz	−0.0784(42)	−0.1698(30)	---	0.16096(97)	−0.0134(16)
E	GHz	36.5593(97)	36.40827(69)		36.40880(24)	
E_J	MHz	1.075(15)	---		---	
F_{bc}	MHz	28.295(13)	28.0573(55)		28.0652(19)	
F_{bcJ}	kHz	---	0.1607(61)		0.1890(45)	
F_{ab}	MHz	79.079(12)	78.9150(21)		78.7240(22)	
F_{abJ}	kHz	---	0.991(32)		1.0105(91)	
F_{abJK}	Hz	---	---		1.253(67)	
N_{A0}/N_{A1}[b]		80	80	20	80/20	21/19/26
rms[c]	kHz	1.3	3.6		40.0	

J	K_a	K_c	v	J	K_a	K_c	v	$\nu_{Obs.}$ MHz	$\nu_{Obs.} - \nu_{Calc.}$ Fit A1	$\nu_{Obs.} - \nu_{Calc.}$ Fit A0/A1 MW	$\nu_{Obs.} - \nu_{Calc.}$ Fit A0/A1 MW+mm
2	0	2	0	1	0	1	0	3108.4942	–0.6	0.2	0.8
4	0	4	0	3	0	3	0	6209.5879	–0.9	0.1	0.7
5	0	5	0	4	0	4	0	7755.0607	–0.6	0.0	0.4
6	0	6	0	5	0	5	0	9295.9683	0.2	0.4	0.2

7	0	7	0	6	0	6	0	10831.4987	0.3	0.1	–0.7
8	0	8	0	7	0	7	0	12360.9594	1.3	1.0	–0.4
9	0	9	0	8	0	8	0	13883.8276	0.2	0.7	–0.9
10	0	10	0	9	0	9	0	15399.8166	–2.6	0.2	–1.1
3	1	3	0	2	1	2	0	4581.6224	–1.3	–1.2	–0.3
4	1	4	0	3	1	3	0	6107.1090	–0.5	–0.7	0.3
5	1	5	0	4	1	4	0	7631.1655	–0.1	–0.7	0.3
6	1	6	0	5	1	5	0	9153.4918	0.9	–0.4	0.7
7	1	7	0	6	1	6	0	10673.8209	0.7	–1.1	0.0
8	1	8	0	7	1	7	0	12191.9296	1.3	–0.6	0.8
9	1	9	0	8	1	8	0	13707.6340	0.0	–1.2	0.9
10	1	10	0	9	1	9	0	15220.8007	–1.3	–0.2	3.1
3	1	2	0	2	1	1	0	4740.8490	–0.6	–0.5	1.1
4	1	3	0	3	1	2	0	6319.2761	–0.6	–0.9	0.9
5	1	4	0	4	1	3	0	7896.0503	–0.3	–1.0	0.7
6	1	5	0	5	1	4	0	9470.6769	–0.5	–1.7	–0.5
7	1	6	0	6	1	5	0	11042.6067	0.4	–1.2	–0.7
8	1	7	0	7	1	6	0	12611.2144	–0.6	–2.4	–2.6
9	1	8	0	8	1	7	0	14175.7923	–1.4	–2.8	–3.6
10	1	9	0	9	1	8	0	15735.5306	0.8	0.8	–0.1
4	2	2	0	3	2	1	0	6215.6765	0.1	–0.2	0.4
5	2	3	0	4	2	2	0	7773.6888	1.2	0.6	1.4
4	2	3	0	3	2	2	0	6204.7701	–0.8	–0.7	–0.5
5	2	4	0	4	2	3	0	7751.8940	0.3	0.3	0.6
7	0	7	0	6	1	5	0	7386.0268	0.0	–3.5	–1.7
8	0	8	0	7	1	6	0	8704.3787	0.1	–2.1	–2.1
9	0	9	0	8	1	7	0	9976.9920	1.0	1.2	–0.4
10	0	10	0	9	1	8	0	11201.0169	0.3	4.8	2.7
2	1	1	0	1	0	1	0	6048.1584	–1.1	7.7	1.1
3	1	2	0	2	0	2	0	7680.5136	–0.7	7.4	1.8
4	1	3	0	3	0	3	0	9339.3629	1.1	7.9	3.4
5	1	4	0	4	0	4	0	11025.8248	1.2	6.4	2.8

[a] All parameters refer to the principal axis system. Watson's S reduction in I^r representation was used.

[b] Number of lines. For the A0/A1 MW+mmw fit, the numbers of A0/A1 MW as well as A0/A1/A01 mm transitions are given. The A01 lines are interstate transitions ($v_t = 0 \leftarrow 1$ or $1 \leftarrow 0$).

[c] Root-mean-squares deviation of the fit.

Table S5: Fitted molecular parameters of the E0 and E1 states of phenyl acetate using theprogram *spfit*; Observed frequencies ($\nu_{obs.}$) of 69 rotational transitions of the E0 species of phenylacetate (Fit E0 in Table S-5a) as well as those of 17 E1 species transitions (Fit E0/E1 in Table S-5a). $\nu_{Obs.} - \nu_{Calc.}$ values (in kHz) are obtained after a fit with the program *spfit*. Observed frequencies $\nu_{Obs.}$ are given in MHz.

**Par.**[a]	**Unit**	**Fit E0**	**Fit E0/E1**	
A	MHz	3621.088(38)	3623.0028(17)	3629.3566(42)
B	MHz	801.872(38)	803.69612(14)	804.83313(50)
C	MHz	748.998(38)	751.02010(13)	749.74731(71)
D_J	kHz	---	8.92044(85)	24.610(31)
D_{JK}	kHz	13.33(25)	−77.728(83)	376.39(35)
D_K	kHz	−9.53(19)	32.29(31)	−47.39(98)
d_1	kHz	---	−0.26530(44)	−27.107(14)
d_2	MHz	---	−9.0950(10)	---
E	GHz	35.6076(68)	22.26975(30)	
E_J	MHz	1.930(38)	---	
F_{bc}	MHz	−28.4735(61)	4.4038(23)	
F_{bcJ}	kHz	0.566(37)	---	
F_{ab}	MHz	76.811(39)	66.9364(73)	
F_{abJ}	kHz	−3.973(73)	---	
D_a	MHz	401.87237(77)	402.01555(90)	380.72713(62)
D_c	MHz	33.6480(40)	20.0845(39)	21.441(75)
D_{aK}	MHz	−0.14046(17)	−0.15446(21)	---
D_{cK}	MHz	−0.7417(96)	25.1754(79)	−31.016(47)
D_{cKK}	MHz	0.1483(16)	−5.5413(12)	
N_{E0}/N_{E1}[b]		69	60	17
rms[c]	kHz	10.6	358.8	

J	K_a	K_c	v	J	K_a	K_c	v	$\nu_{Obs.}$ MHz	$\nu_{Obs.} - \nu_{Calc.}$ Fit E0	$\nu_{Obs.} - \nu_{Calc.}$ Fit E0/E1
5	0	5	0	4	0	4	0	7751.4642	3.3	56.4
6	0	6	0	5	0	5	0	9291.0819	3.4	87.7
7	0	7	0	6	0	6	0	10824.9537	3.6	102.6

8	0	8	0	7	0	7	0	12352.2719	–0.5	62.1
9	0	9	0	8	0	8	0	13872.3882	–13.4	–93.7
5	1	4	0	4	1	3	0	7846.2794	–1.3	104.3
6	1	5	0	5	1	4	0	9431.3367	0.3	78.3
7	1	6	0	6	1	5	0	11012.5809	0.4	28.9
8	1	7	0	7	1	6	0	12588.1826	2.5	–10.9
9	1	8	0	8	1	7	0	14157.5752	2.2	–0.3
6	1	6	0	5	1	5	0	9193.8192	6.9	–39.8
7	1	7	0	6	1	6	0	10705.5461	0.2	2.3
8	1	8	0	7	1	7	0	12217.7159	–16.6	102.3
6	2	4	0	5	2	3	0	9313.8829	4.3	51.0
7	2	5	0	6	2	4	0	10866.8120	3.8	–170.3
8	2	6	0	7	2	5	0	12421.1218	–5.4	–334.2
2	1	1	0	1	0	1	0	6363.7671	6.3	–126.1
3	1	2	0	2	0	2	0	7939.8172	7.0	–52.9
4	1	3	0	3	0	3	0	9542.8732	2.7	37.4
5	1	4	0	4	0	4	0	11182.1519	–0.2	116.4
6	1	5	0	5	0	5	0	12862.0247	–3.0	138.5
7	1	6	0	6	0	6	0	14583.5246	–5.0	80.7
8	1	7	0	7	0	7	0	16346.7536	–6.0	–32.7
9	1	8	0	8	0	8	0	18152.0549	–5.4	–97.1
10	1	9	0	9	0	9	0	20000.1015	6.7	127.1
2	2	0	0	1	1	0	0	12044.4987	1.5	–296.8
2	2	1	0	2	1	2	0	8143.1451	–13.1	–366.2
3	2	2	0	3	1	3	0	8161.3736	–1.7	–128.1
4	2	3	0	4	1	4	0	8206.3684	7.8	118.0
5	2	4	0	5	1	5	0	8287.3646	9.6	295.0
6	2	5	0	6	1	6	0	8408.2908	3.8	333.4
7	2	6	0	7	1	7	0	8568.9705	–7.2	212.8
8	2	7	0	8	1	8	0	8767.0855	–13.0	13.1
9	2	8	0	9	1	9	0	8998.7679	13.3	–24.9
2	2	1	0	1	1	1	0	11244.5069	–7.9	–332.4
3	2	2	0	2	1	2	0	12800.5087	4.2	–111.8
3	2	1	0	2	1	1	0	13586.6575	–2.8	13.7

[a] All parameters refer to the principal axis system. Watson's S reduction in I^r representation was used.

[b] Number of lines.

[c] Root-mean-squares deviation of the fit.

Table S6: Fitted molecular parameters of the A0 and E0 states of phenyl acetate using theprogram *XIAM*; Observed frequencies ($\nu_{obs.}$) of 80 rotational transitions of the A0 species and 69 transitions of the E0 species of phenyl acetate. $\nu_{Obs.} - \nu_{Calc.}$ values (in MHz) are obtained after a fit with the program *XIAM*. Observed frequencies $\nu_{Obs.}$ are given in MHz.

**Par.**[a]	**Unit**	**Fit A0/E0**	**Calc.**[b]
A	MHz	3632.65(48)	3592.4
B	MHz	804.71(15)	813.9
C	MHz	750.19(17)	744.3
D_J	kHz	8.17(98)	
D_{JK}	MHz	0.4312(86)	
d_1	kHz	−8.52(43)	
d_2	kHz	−37.74(30)	
V_3	cm^{-1}	136.44(37)	113.2
$\angle(i,a)$	°	24.61(75)	23.2
$\angle(i,b)$	°	76.37(40)	77.1
$\angle(i,c)$	°	69.92(60)	71.0
N_{A0}/N_{E0}[c]		80/69	
rms[d]	kHz	6872.0	

							$\nu_{Obs.}$	$\nu_{Obs.} - \nu_{Calc.}$
J	K_a	K_c	J	K_a	K_c		MHz	Fit A0/E0
2	0	2	1	0	1	A1	3108.4942	–0.46
4	0	4	3	0	3	A1	6209.5879	–0.73
5	0	5	4	0	4	A1	7755.0607	–0.87
6	0	6	5	0	5	A1	9295.9683	–1.12
7	0	7	6	0	6	A1	10831.4987	–1.49
8	0	8	7	0	7	A1	12360.9594	–1.81
9	0	9	8	0	8	A1	13883.8276	–1.61
10	0	10	9	0	9	A1	15399.8166	0.06
3	1	3	2	1	2	A1	4581.6224	1.43
4	1	4	3	1	3	A1	6107.1090	1.16
5	1	5	4	1	4	A1	7631.1655	0.44
6	1	6	5	1	5	A1	9153.9180	–0.16
7	1	7	6	1	6	A1	10673.8209	–1.60
8	1	8	7	1	7	A1	12191.9296	–2.03
9	1	9	8	1	8	A1	13707.6340	–0.99

10	1	10	9	1	9	A1	15220.8007	2.79
3	1	2	2	1	1	A1	4740.8490	–1.04
4	1	3	3	1	2	A1	6319.2761	–0.62
5	1	4	4	1	3	A1	7896.0503	0.19
6	1	5	5	1	4	A1	9470.6769	1.20
7	1	6	6	1	5	A1	11042.6067	2.05
8	1	7	7	1	6	A1	12611.2144	2.21
9	1	8	8	1	7	A1	14175.7923	1.08
10	1	9	9	1	8	A1	15735.5306	–2.06
4	2	2	3	2	1	A1	6215.6765	3.12
5	2	3	4	2	2	A1	7773.6888	3.60
4	2	3	3	2	2	A1	6204.7701	2.75
5	2	4	4	2	3	A1	7751.8940	3.08
7	0	7	6	1	5	A1	7386.0268	0.21
8	0	8	7	1	6	A1	8704.3787	–3.65
9	0	9	8	1	7	A1	9976.9920	–7.47
10	0	10	9	1	8	A1	11201.0169	–8.48
2	1	1	1	0	1	A1	6048.1584	–5.22
3	1	2	2	0	2	A1	7680.5136	–5.80
4	1	3	3	0	3	A1	9339.3629	–5.80
5	1	4	4	0	4	A1	11025.8248	–4.88
6	1	5	5	0	5	A1	12741.4407	–2.81

[a] All parameters refer to the principal axis system. Watson's S reduction in I^r representation was used.

[b] Values of the equilibrium structure calculated at the MP2/6-311++G(d,p) level of theory.

[c] Number of lines.

[d] Root-mean-squares deviation of the fit.

Table S7. Observed frequencies ($\nu_{obs.}$) of 240 rotational transitions of phenyl acetate belonging to the four sub-states A0, A1, E0, and E1. $\nu_{Obs.} - \nu_{Calc.}$ values (in kHz) are obtained after a fit with the program *spfit*. Observed frequencies $\nu_{Obs.}$ are given in MHz.

J	K_a	K_c	*v*	*J*	K_a	K_c	*v*	$\nu_{Obs.}$	$\nu_{Obs.} - \nu_{Calc.}$
10	10	0	0	9	9	0	0	69860.27	55.7
10	10	0	1	9	9	0	1	69973.31	–31.7
11	10	1	0	10	9	1	0	71367.20	62.0
11	10	1	1	10	9	1	1	71539.35	–71.5
12	10	2	0	11	9	2	0	72882.86	52.3
12	10	2	1	11	9	2	1	73103.17	–46.2
9	9	0	0	8	8	0	0	62463.90	39.6
9	9	0	1	8	8	0	1	62690.04	–4.6
10	9	1	0	9	8	1	0	63899.06	20.7
10	9	1	1	9	8	1	1	64294.65	22.1
11	9	2	0	10	8	2	0	65370.52	–15.8
11	9	2	1	10	8	2	1	65889.22	–35.3
13	9	4	0	12	8	4	0	68381.99	–8.9
13	9	4	1	12	8	4	1	69051.58	12.2
14	9	5	0	13	8	5	0	69910.62	–15.5
14	9	5	1	13	8	5	1	70620.86	7.3
15	9	6	0	14	8	6	0	71449.86	–81.7
15	9	6	1	14	8	6	1	72183.24	19.9
16	9	7	0	15	8	7	0	72997.60	–90.4
16	9	7	1	15	8	7	1	73739.29	–23.5
11	8	3	1	10	7	3	1	60427.38	94.5
12	8	4	0	11	7	4	0	60400.58	–54.6
12	8	4	1	11	7	4	1	62031.47	91.9
13	8	5	0	12	7	5	0	61964.70	–13.4
13	8	5	1	12	7	5	1	63616.43	97.5
14	8	6	1	13	7	6	1	65186.53	65.5
15	8	7	1	14	7	7	1	66744.70	21.4
16	8	8	1	15	7	8	1	68293.01	34.4
17	8	9	1	16	7	9	1	69832.76	17.0
18	8	10	1	17	7	10	1	71364.89	–39.3
19	8	11	1	18	7	11	1	72890.09	–64.8
17	7	10	0	16	6	10	0	59624.34	–27.9
18	7	11	0	17	6	11	0	61010.03	–133.8
18	7	12	0	17	6	12	0	61010.49	–143.7
19	7	12	0	18	6	12	0	62386.59	–53.2
19	7	13	0	18	6	13	0	62387.42	–148.5
20	7	13	0	19	6	13	0	63751.88	45.4
20	7	14	0	19	6	14	0	63753.51	–76.4
21	7	14	0	20	6	14	0	65104.52	186.2

21	7	15	0	20	6	15	0	65107.61	81.4
15	8	7	0	14	6	8	1	65121.42	15.3
16	8	8	0	15	6	10	1	66709.00	–48.0
17	8	9	0	16	6	11	1	68301.05	14.3
18	8	10	0	17	6	12	1	69896.57	–0.4
19	8	11	0	18	6	13	1	71495.17	32.0
20	8	12	0	19	6	14	1	73096.39	–35.2
14	7	7	1	13	7	6	0	60090.62	83.4
14	7	8	1	13	7	7	0	60090.62	83.3
15	7	8	1	14	7	7	0	61601.87	66.0
15	7	9	1	14	7	8	0	61601.87	65.7
16	7	9	1	15	7	8	0	63104.76	55.8
16	7	10	1	15	7	9	0	63104.76	55.1
17	7	10	1	16	7	9	0	64599.96	18.9
17	7	11	1	16	7	10	0	64599.96	17.2
18	7	11	1	17	7	10	0	66087.71	–27.7
18	7	12	1	17	7	11	0	66087.71	–31.9
19	7	12	1	18	7	11	0	67567.96	–21.3
19	7	13	1	18	7	12	0	67567.96	–30.9
20	7	13	1	19	7	12	0	69040.32	1.2
20	7	14	1	19	7	13	0	69040.32	–19.9
21	7	14	1	20	7	13	0	70504.25	17.8
21	7	15	1	20	7	14	0	70504.25	–27.0
22	7	15	1	21	7	14	0	71959.17	52.5
22	7	16	1	21	7	15	0	71959.17	–40.0
23	7	16	1	22	7	15	0	73404.44	58.1
23	7	17	1	22	7	16	0	73404.44	–127.0
2	0	2	0	1	0	1	0	3108.4942	0.7
4	0	4	0	3	0	3	0	6209.5879	0.6
5	0	5	0	4	0	4	0	7755.0607	0.4
6	0	6	0	5	0	5	0	9295.9683	0.3
7	0	7	0	6	0	6	0	10831.4987	–0.6
8	0	8	0	7	0	7	0	12360.9594	–0.3
9	0	9	0	8	0	8	0	13883.8276	–1.1
10	0	10	0	9	0	9	0	15399.8166	–2.0
3	1	3	0	2	1	2	0	4581.6224	–0.1
4	1	4	0	3	1	3	0	6107.1090	0.5
5	1	5	0	4	1	4	0	7631.1655	0.5
6	1	6	0	5	1	5	0	9153.4918	0.7
7	1	7	0	6	1	6	0	10673.8209	–0.1
8	1	8	0	7	1	7	0	12191.9296	0.3
9	1	9	0	8	1	8	0	13707.6340	–0.2
10	1	10	0	9	1	9	0	15220.8007	1.2
3	1	2	0	2	1	1	0	4740.8490	0.7
4	1	3	0	3	1	2	0	6319.2761	0.6
5	1	4	0	4	1	3	0	7896.0503	0.4

6	1	5	0	5	1	4	0	9470.6769	–0.5
7	1	6	0	6	1	5	0	11042.6067	–0.5
8	1	7	0	7	1	6	0	12611.2144	–2.1
9	1	8	0	8	1	7	0	14175.7923	–3.0
10	1	9	0	9	1	8	0	15735.5306	0.5
4	2	2	0	3	2	1	0	6215.6765	0.4
5	2	3	0	4	2	2	0	7773.6888	1.4
4	2	3	0	3	2	2	0	6204.7701	–0.3
5	2	4	0	4	2	3	0	7751.8940	0.7
7	0	7	0	6	1	5	0	7386.0268	–0.5
8	0	8	0	7	1	6	0	8704.3787	–1.1
9	0	9	0	8	1	7	0	9976.9920	0.0
10	0	10	0	9	1	8	0	11201.0169	1.6
2	1	1	0	1	0	1	0	6048.1584	0.9
3	1	2	0	2	0	2	0	7680.5136	1.3
4	1	3	0	3	0	3	0	9339.3629	2.7
5	1	4	0	4	0	4	0	11025.8248	2.0
6	1	5	0	5	0	5	0	12741.4407	0.8
7	1	6	0	6	0	6	0	14488.0778	–1.4
8	1	7	0	7	0	7	0	16267.7935	–2.9
9	1	8	0	8	0	8	0	18082.6308	–1.2
10	1	9	0	9	0	9	0	19934.3311	–2.4
11	1	10	0	10	0	10	0	21824.0082	3.7
2	2	0	0	1	1	0	0	11663.0142	0.6
3	2	1	0	2	1	1	0	13161.6347	0.9
4	2	2	0	3	1	2	0	14636.4606	–1.0
5	2	3	0	4	1	3	0	16090.8723	–1.2
6	2	4	0	5	1	4	0	17529.1857	–1.4
7	2	5	0	6	1	5	0	18956.6266	–2.0
8	2	6	0	7	1	6	0	20379.2607	–1.6
9	2	7	0	8	1	7	0	21803.8708	0.1
10	2	8	0	9	1	8	0	23237.7920	4.2
2	2	1	0	1	1	1	0	11715.0204	1.7
3	2	2	0	2	1	2	0	13315.4571	0.5
4	2	3	0	3	1	3	0	14938.6052	0.7
5	2	4	0	4	1	4	0	16583.3903	1.0
6	2	5	0	5	1	5	0	18248.5244	0.5
7	2	6	0	6	1	6	0	19932.4812	0.8
8	2	7	0	7	1	7	0	21633.4639	1.5
9	2	8	0	8	1	8	0	23349.3822	3.9
3	2	1	0	3	1	3	0	8739.2871	0.0
4	2	2	0	4	1	4	0	8847.8553	0.7
5	2	3	0	5	1	5	0	8990.3760	–1.1
6	2	4	0	6	1	6	0	9171.2491	–0.3
7	2	5	0	7	1	7	0	9395.5471	–0.4
8	2	6	0	8	1	8	0	9668.8574	–1.6

9	2	7	0	9	1	9	0	9997.0468	–3.0
10	2	8	0	10	1	10	0	10385.9660	3.4
3	2	2	0	3	1	2	0	8415.3275	–5.0
4	2	3	0	4	1	3	0	8300.8285	1.1
5	2	4	0	5	1	4	0	8156.6692	–1.7
6	2	5	0	6	1	5	0	7982.2909	–2.2
3	2	1	0	3	1	3	0	8739.2859	–1.2
4	2	2	0	4	1	4	0	8847.8556	1.0
5	2	3	0	5	1	5	0	8990.3760	–1.1
6	2	4	0	6	1	6	0	9171.2500	0.6
7	2	5	0	7	1	7	0	9395.5470	–0.5
8	2	6	0	8	1	8	0	9668.8569	–2.1
8	1	7	0	7	2	5	0	4697.1924	–2.7
9	1	8	0	8	2	6	0	6407.7480	–1.6
11	1	10	0	10	2	8	0	9787.2322	0.3
3	1	2	1	2	0	2	1	7690.4720	6.0
4	1	3	1	3	0	3	1	9358.0513	5.3
5	1	4	1	4	0	4	1	11057.8825	1.6
6	1	5	1	5	0	5	1	12792.5630	–3.2
7	1	6	1	6	0	6	1	14565.1298	–9.8
8	1	7	1	7	0	7	1	16378.9919	–9.9
9	1	8	1	8	0	8	1	18237.8357	11.4
2	2	0	1	1	1	0	1	11674.5391	–6.8
3	2	1	1	2	1	1	1	13187.6276	6.8
2	2	0	1	2	1	2	1	8672.1067	0.7
2	2	1	1	1	1	1	1	11727.2715	–2.9
3	2	2	1	2	1	2	1	13344.6781	–1.9
3	2	2	1	3	1	2	1	8440.7634	–6.5
4	2	3	1	4	1	3	1	8355.3119	–3.3
5	2	4	1	5	1	4	1	8261.1730	2.0
4	2	2	1	4	1	4	1	8896.1206	1.0
5	2	3	1	5	1	5	1	9078.6066	7.3
6	2	4	1	6	1	6	1	9317.2543	11.1
7	2	5	1	7	1	7	1	9616.5305	–8.0
8	0	8	1	7	1	6	1	8685.8878	2.5
5	0	5	2	4	0	4	2	7751.4642	1.1
6	0	6	2	5	0	5	2	9291.0819	–1.7
7	0	7	2	6	0	6	2	10824.9537	–1.4
8	0	8	2	7	0	7	2	12352.2719	0.4
9	0	9	2	8	0	8	2	13872.3882	2.6
5	1	5	2	4	1	4	2	7681.4597	–2.1
6	1	6	2	5	1	5	2	9193.8192	6.4
7	1	7	2	6	1	6	2	10705.5461	5.7
8	1	8	2	7	1	7	2	12217.7159	–14.9
5	1	4	2	4	1	3	2	7846.2794	2.1
6	1	5	2	5	1	4	2	9431.3367	1.7

7	1	6	2	6	1	5	2	11012.5809	–1.6
8	1	7	2	7	1	6	2	12588.1826	–1.5
9	1	8	2	8	1	7	2	14157.5752	0.8
2	1	1	2	1	0	1	2	6363.7671	7.6
3	1	2	2	2	0	2	2	7939.8172	3.1
4	1	3	2	3	0	3	2	9542.8732	–2.2
5	1	4	2	4	0	4	2	11182.1519	–2.5
6	1	5	2	5	0	5	2	12862.0247	–1.5
7	1	6	2	6	0	6	2	14583.5246	–0.5
8	1	7	2	7	0	7	2	16346.7536	–0.6
9	1	8	2	8	0	8	2	18152.0549	–2.2
10	1	9	2	9	0	9	2	20000.1015	4.1
7	0	7	2	6	1	5	2	7254.0108	–1.6
8	0	8	2	7	1	6	2	8593.6998	–1.6
9	0	9	2	8	1	7	2	9877.9060	3.1
2	2	0	2	1	1	0	2	12044.4987	6.0
2	2	1	2	1	1	1	2	11244.5035	–0.8
3	2	1	2	3	1	3	2	9766.2308	–0.6
6	2	4	2	5	2	3	2	9313.8829	10.4
7	2	5	2	6	2	4	2	10866.8120	10.0
8	2	6	2	7	2	5	2	12421.1218	–9.7
4	2	2	2	4	1	4	2	9810.6165	–4.5
5	2	3	2	5	1	5	2	9890.6759	–3.8
6	2	4	2	6	1	6	2	10010.7377	–1.8
7	2	5	2	7	1	7	2	10172.0048	3.7
8	2	6	2	8	1	8	2	10375.4088	6.9
9	2	7	2	9	1	9	2	10623.4504	2.5
10	2	8	2	10	1	10	2	10921.0265	–14.5
11	2	9	2	11	1	11	2	11275.5630	6.1
2	2	1	2	2	1	2	2	8143.1451	4.6
3	2	2	2	3	1	3	2	8161.3736	–1.2
4	2	3	2	4	1	4	2	8206.3684	–1.8
5	2	4	2	5	1	5	2	8287.3646	–0.7
6	2	5	2	6	1	6	2	8408.2908	1.4
7	2	6	2	7	1	7	2	8568.9705	3.2
8	2	7	2	8	1	8	2	8767.0855	2.7
9	2	8	2	9	1	9	2	8998.7679	–3.4
2	2	1	2	1	1	1	2	11244.5069	2.7
3	2	2	2	2	1	2	2	12800.5087	–6.4
3	2	1	2	2	1	1	2	13586.6575	–2.3
2	2	0	2	2	1	1	2	8929.5822	11.1
3	2	1	2	3	1	2	2	8903.2291	1.0
4	2	2	2	4	1	3	2	8850.8349	–6.6
5	2	3	2	5	1	4	2	8766.0758	–8.8
6	2	4	2	6	1	5	2	8648.6208	–1.4
7	2	5	2	7	1	6	2	8502.8501	8.4

8	2	6	2	8	1	7	2	8335.7881	–1.0
4	1	4	2	3	0	3	2	8583.0909	–4.9
5	1	5	2	4	0	4	2	10057.5530	–6.3
6	1	6	2	5	0	5	2	11499.9080	–0.9
7	1	7	2	6	0	6	2	12914.3708	5.1
3	1	2	3	2	0	2	3	7930.9383	–0.1
4	1	3	3	3	0	3	3	9544.9891	–2.7
5	1	4	3	4	0	4	3	11200.7613	6.4
6	1	5	3	5	0	5	3	12903.0873	–4.5
7	1	6	3	6	0	6	3	14653.7840	1.0
2	2	0	3	1	1	0	3	12037.6303	–3.8
3	2	1	3	2	1	1	3	13595.6562	2.0
2	2	1	3	1	1	1	3	11279.2188	0.7
3	2	2	3	2	1	2	3	12851.6183	–0.1
2	2	1	3	2	1	2	3	8177.3047	–0.6
2	2	0	3	2	1	1	3	8921.3300	3.7
3	2	1	3	3	1	2	3	8907.7525	–1.9
